# Re-comparing of three different epidemic seasons of bronchiolitis: different prophylaxis approaches

**DOI:** 10.1186/s13052-018-0593-7

**Published:** 2018-12-12

**Authors:** Simonetta Picone, Adele Fabiano, Davide Roma, Federico Di Palma, Piermichele Paolillo

**Affiliations:** 1 Neonatology-NICU Casilino General Hospital, via Casilina 1049, 00169 Rome, Italy; 2 Unit of Pediatrics and Neonatology San Camillo de Lellis Hospital, viale Kennedy, 02100 Rieti, Italy; 3 Medical School La Sapienza University, piazzale Aldo Moro 5, 00185 Rome, Italy

**Keywords:** Bronchiolitis, Prophylaxis, Preterm, Palivizumab

## Abstract

During the last epidemic season of bronchiolitis (S2, years 2016–2017) we performed a single Centre analysis in inborn infant of 30^+ 0^–32^+ 6^ gestational age and age < 12 months who did not receive prophylaxis with palivizumab (PLV), in light of the current AIFA (Italian Drug Agency) guidelines restricting the time of the prophylaxis to those born < 30 weeks of gestational age. During that epidemic season, we observed a rising trend of bronchiolitis-related hospitalization and an increased rate of mechanical ventilation in preterm child compared to the previous one (S1, years 2015–2016) during which infants of this same gestational age received palivizumab (PLV) prophylaxis, according to the 2015 Italian Guidelines.

In light of the revised AIFA guidelines (November 2017), allowing once again prophylaxis with PLV in infants of > 30 weeks gestational age, we decided to repeat our observation during the last epidemic season (S3, years 2017–2018), in order to compare ours infants of 30^+ 0^–32^+ 6^ gestational age with preterm of the same gestational age born in our unit in the previous seasons (S1 and S2), to evaluate the clinical impact of the different prophylaxis approaches.

The new observation confirmed the clinical efficacy of PLV in this delicate group of newborns in preventing almost completely new episodes of bronchiolitis. Of the 6 newborns who developed bronchiolitis, 4 had received only a single dose of PLV, providing suboptimal protection, before the onset of bronchiolitis; furthermore 3 developed a mild form allowing to be treated at home.

## Dear Editor,

During the last epidemic season of bronchiolitis, we performed a single center, retrospective analysis in our infant with GA between 30^+ 0^ and 32^+ 6^ weeks and age < 12 months to compare frequency and characteristics of bronchiolitis and bronchiolitis-related hospitalization, in two consecutive epidemic seasons (S1 vs S2). In S1 (May 1st 2015 to March 31st 2016) 35 newborns had received prophylaxis with PLV (according to 2015 AIFA guidelines allowing prophylaxis to all newborn with a < 35 week of gestational age and with less of 6 months at the start of epidemic season, identified in in Italy from October to April); in S2 (May 1st, 2016 to March 31st, 2017) 47 newborns of the same gestational age did not receive prophylaxis due to a modification of AIFA indications for prophylaxis limiting it only to newborns < 30 weeks of gestational age (October 2016) [1]. In S2 vs. S1, we observed a rising trend in rates of bronchiolitis and bronchiolitis-related hospitalization and of morbidity with an increase in the rate of mechanical ventilation and a reduction in the age of hospitalization for bronchiolitis in S2 [2].

In November 2017, AIFA indications authorized again prophylaxis for preterm ≤35 weeks of GE and with less of 6 months at the start of epidemic season [3]. Therefore, infants with GE 30^+ 0^ to 32^+ 6^ regularly received PLV in the S3 epidemic season (S3, years 2017–2018 from May 1st, 2017 to May 31st, 2018).

In order to assess, any potential clinical impact of the different path of prophylaxis, we then compared the incidence of bronchiolitis and the characteristics of the bronchiolitis-related hospitalization between S1, S2 and S3 in inborn infants of 30^+ 0^–32^+ 6^ weeks gestational age. Our data were reported in Table 1.

**Table 1:** Clinical characteristic of three groups (WGE = week of gestational age)

	S1 (2015–2016)	S2 (2016–2017)	S3 (2017–2018)	*P* value^*^
Newborn with WGE 30^+ 0^–32^+ 6^	35	47	56	
Mean gestational age	30,9	31	31,5	
Newborn with WGE < 31 sett (30^+ 0^–30^+ 6^)	16/35 (45,7%)	18/47 (38,3%)	16/56 (28,57%)	*P* = 0.29
Newborn treated with PLV	27	2	56	*P* < 0,00001
Number of bronchiolitis	6/35 (17%)	12/47 (26%)	6/56 (10,7%)	*P* =0.048
Hospitalized for bronchiolitis	3/6 (50%)	6/12 (50%)	3^a^/6 (50%)	*P* = 1
Absolute percentage of admissions	3/35 (8,6%)	6/47 (12,8%)	3/56 (5,3%)	*P* =0.184
Intubation at birth	3/3 (100%)	4/6 (66,7%)	0/3 (0%)	*P* = 0,16
Age at the admission (Months)	7	4.3	3^b^	
Intubation at admission	1/3 (33,3%)	2/6 (33,3%)	1^c^/3 (33,3%)	*P* = 1

^*^all statistical analysis was performed comparing S2 with S3

^a^All 3 preterm had received only one dose of palivizumab before having bronchiolitis

^b^All 3 preterm had received only one dose of palivizumab before having bronchiolitis and were born in full epidemic season

^c^newborn who had received only one dose of palivizumab

During the 2017–2018 epidemic season, according to the new AIFA recommendations for 2017, all 56 infants of 30^+ 0^–32^+ 6^ GE underwent PLV prophylaxis. The percentage of bronchiolitis declined from 26% (12/47) in S2 to 10,7% in S3 (6/56). Hospital admissions for bronchiolitis were 3/6 (50%), like the previous season, but all the three admissions with one treated at home had received only one dose of PLV before contracting bronchiolitis, hence without a full course of adequate prophylaxis; additional 2 children developed a mild form and were treated at home. Although there is a slight difference in the average gestational age of newborns between S2 and S3 (with a higher GE in S3) we believe that this difference does not affect the results of the study, particularly the major EG of S3 is not the factor determining the lower incidence of bronchiolitis. In fact, in S2, 10 of 12 infants with bronchiolitis had a lower EG (30–30^+ 6^), while in S3 of 6 infants with bronchiolitis only 1 had the lowest EG (30–30^+ 6^): this shows that in the epidemic season without prophylaxis (S2) as expected, the most premature infants are affected, while in the epidemic season in which prophylaxis is carried out (S3) the youngest are protected in almost all cases and those affected by bronchiolitis are those with high gestational age.

The mean chronological age at hospitalization for bronchiolitis was 3 months, but they were all newborns (4) who had received only one dose of PLV and three of them were born in full epidemic season. In S3 only one newborn needed intubation during the hospitalization for bronchiolitis and he had received only one dose of PLV.

Overall, our observations confirm the efficacy of prophylaxis with PLV in infants 30–32 weeks GE, which in S3 were protected by bronchiolitis in almost all cases and indicating that the most severe forms requiring hospitalization and intubation would have potentially been equal to zero if the only hospitalized and intubated infant had time to complete the PLV treatment cycle.

Although such data cannot be generalized, our personal experience suggests that prophylaxis with PLV in preterm of this gestational age is a good medical practice and should not be further modified.

## Abbreviations

GA: Gestational age
PLV: Palivizumab
WGA: Week of gestational age
